# Complete mitogenome sequences of smooth hammerhead sharks, *Sphyrna zygaena*, from the eastern and western Atlantic

**DOI:** 10.1080/23802359.2017.1390421

**Published:** 2017-11-10

**Authors:** Derek S. Guy, Cassandra L. Ruck, Jose V. Lopez, Mahmood S. Shivji

**Affiliations:** Save Our Seas Shark Research Center and Guy Harvey Research Institute, Department of Biological Sciences, Nova Southeastern University, Dania Beach, FL, USA

**Keywords:** *Sphyrna zygaena*, Vulnerable, eastern Atlantic, western Atlantic, mitochondrial genome

## Abstract

We report the first mitogenome sequences of the circumglobally distributed, highly mobile, smooth hammerhead shark, *Sphyrna zygaena*, from the eastern and western Atlantic. Both genomes were 16,729 bp long with 13 protein-coding genes, two rRNAs, 22 tRNAs and a non-coding control region. The two Atlantic shark sequences differ from each other by 13 SNPs, and by 43 and 44 SNPs from the published mitogenome of an *S. zygaena* specimen from the eastern Pacific Ocean. The cross-Atlantic mitogenome sequences reported here provide a resource to assist with population genetics studies of this widely exploited species of conservation concern.

Similar to other large-bodied elasmobranch species, the smooth hammerhead shark, *Sphyrna zygaena* (Sphyrnidae), has suffered population declines due to over-exploitation in many parts of its global range. This species is listed as Vulnerable on the International Union for the Conservation of Nature (IUCN) Red List (Casper et al. 2005) and included on CITES Appendix II. Information on population dynamics of this species is severely lacking. Bolaño-Martínez et al. (2016) reported the first mitogenome for this species from a specimen caught in the eastern Pacific (RefSeq: NC_025778.1). To complement this resource, we present the first mitogenome sequences of two individuals from different geographic regions of the Atlantic Ocean. These sequences will serve as informative references for future population genetics studies of *S. zygaena* and elasmobranch phylogenetics.

The two specimens were from a male sampled in 2011 in the eastern Atlantic off the Azores (geospatial coordinates: 38.615967, −28.793667) and a female sampled in 2016 in the western Atlantic off the coast of Maryland, USA (geospatial coordinates: 38.216667, −75.033333). Fin clip samples (accession numbers: OC-448 and OC-462) were stored in 100% ethanol at the Save Our Seas Shark Research Center, Nova Southeastern University. DNA extractions were performed using a QIAGEN DNeasy Blood & Tissue Extraction Kit (Qiagen Inc., Valencia, CA) and amplified via long PCR in four fragments using KAPA HiFi HotStart ReadyMix PCR (Kapa Biosystems, Boston, MA). The amplicons were purified with Agencourt^®^ AMPure XP Beads (Beckman Coulter Life Sciences, Indianapolis, IN). Libraries were prepared for sequencing on an Illumina MiSeq system with paired-end reads (2 × 250 bp) using a Nextera XT DNA Library Preparation Kit (Illumina, San Diego, CA). The resulting FASTQ files were quality filtered using the Minoche protocol (Eren et al. 2013). Assisted assemblies were performed with Bowtie2 (Langmead and Salzberg 2012) using the eastern Pacific *S. zygaena* published sequence as the reference index. The final assemblies were annotated with MitoAnnotator (Iwasaki et al. 2013).

The mitogenomes of both Atlantic *S. zygaena* individuals (gb: MF983538 and MF983539) were 16,729 bp in length containing 13 protein-coding genes, 22 tRNAs, two rRNAs, and a non-coding control region, with an identical gene organization to other elasmobranch mitogenomes. Similar to other elasmobranchs, the *COII* and *ND4* genes contain incomplete stop codons. A MUSCLE alignment with the five other published Sphyrnidae mitochondrial genomes and the blacktip reef shark, *Carcharhinus melanopterus* (as the outgroup), was performed in Genious^®^ 7.1.9 (http://geneious.com, Kearse et al. 2012). The two Atlantic *S. zygaena* individuals were differentiated by 13 SNPs (99.92% identity). The eastern and western Atlantic individuals were differentiated by 43 and 44 SNPs (∼99.74% identity), respectively from the eastern Pacific *S. zygaena* sequence. A Bayesian tree (Figure 1) was constructed with default parameters in MrBayes 3.2. (Huelsenbeck and Ronquist 2001; Ronquist and Huelsenbeck 2003) using the GTR + G substitution model, which was determined as the best substitution model by the Bayesian Information Criterion in jModelTest 2.1.4 (Darriba et al. 2012).

**Figure 1.**
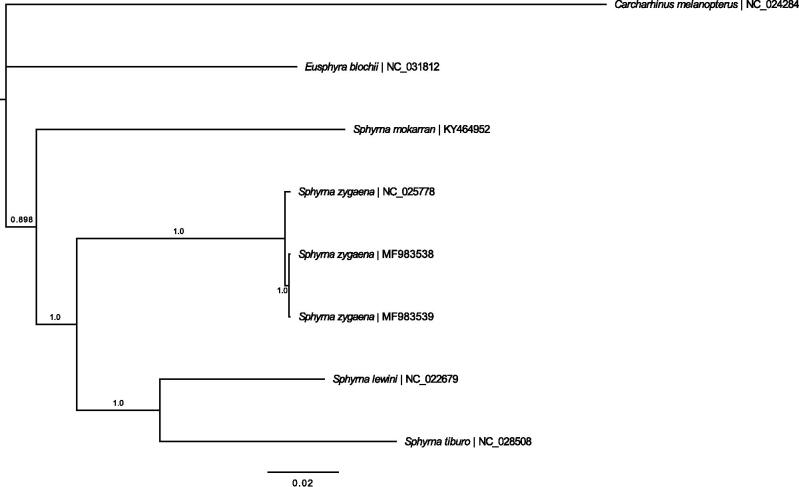
The Bayesian tree generated via MrBayes 3.2 with all published Sphyrnidae mitogenomes and a Carcharhinid species (*C. melonopterus*) as the outgroup. Posterior probabilities of the clades and NCBI Accession numbers are shown.
